# The Evolution and Ecology of Oxidative and Antioxidant Status: A Comparative Approach in African Mole-Rats

**DOI:** 10.3390/antiox12081486

**Published:** 2023-07-25

**Authors:** Paul. J. Jacobs, Daniel W. Hart, Hana N. Merchant, Cornelia Voigt, Nigel C. Bennett

**Affiliations:** 1 Department of Zoology and Entomology, University of Pretoria, Pretoria 0002, South Africa; daniel.hart@zoology.up.ac.za; 2 Department of Biological Sciences, School of Life and Environmental Sciences, Royal Holloway University of London, Egham Hill, Egham, Surrey TW20 0EX, UK; hana.merchant.2020@live.rhul.ac.uk; 3 Mammal Research Institute, Department of Zoology and Entomology, University of Pretoria, Pretoria 0002, South Africa; cvoigt@zoology.up.ac.za (C.V.); ncbennett@zoology.up.ac.za (N.C.B.)

**Keywords:** oxidative stress, oxidative status, longevity, ageing, maximum lifespan potential, sociality, metabolism, aridity, antioxidants, reactive oxygen species

## Abstract

The naked mole-rat of the family Bathyergidae has been the showpiece for ageing research as they contradict the traditional understanding of the oxidative stress theory of ageing. Some other bathyergids also possess increased lifespans, but there has been a remarkable lack of comparison between species within the family Bathyergidae. This study set out to investigate how plasma oxidative markers (total oxidant status (TOS), total antioxidant capacity (TAC), and the oxidative stress index (OSI)) differ between five species and three subspecies of bathyergids, differing in their maximum lifespan potential (MLSP), resting metabolic rate, aridity index (AI), and sociality. We also investigated how oxidative markers may differ between captive and wild-caught mole-rats. Our results reveal that increased TOS, TAC, and OSI are associated with increased MLSP. This pattern is more prevalent in the social-living species than the solitary-living species. We also found that oxidative variables decreased with an increasing AI and that wild-caught individuals typically have higher antioxidants. We speculate that the correlation between higher oxidative markers and MLSP is due to the hypoxia-tolerance of the mole-rats investigated. Hormesis (the biphasic response to oxidative stress promoting protection) is a likely mechanism behind the increased oxidative markers observed and promotes longevity in some members of the Bathyergidae family.

## 1. Introduction

The ‘Great oxidation event’ (some 2.45 billion years ago) saw the Earth’s atmosphere and the shallow ocean experience a significant rise in the amount of oxygen, likely a result of the evolution of photosynthesis by Archean, anaerobic cyanobacteria [1,2,3]. The increase in the oxygen composition of the atmosphere acted as an intense selective pressure on anaerobic organisms, paving the way forward to a world dominated by aerobic organisms [1,4,5]. However, despite the increased concentration of oxygen providing a more energy-efficient means for metabolism, it also came with its own set of problems, the most significant being the formation of oxygen4-derived free radicals or non-radical reactive oxygen species, typically known as reactive oxygen species (ROS) [6,7,8]. ROS can harm biological systems [9,10,11] and likely mediates ageing [12,13]. Thus, the study of ROS and oxidative stress {the balance between oxidative damage and antioxidant (compounds and mechanisms that inhibit oxidation) measures} has been of vital interest for investigating life-history ecology and evolution [4,14,15,16,17,18].

Ageing has been described as gradual fitness loss due to detrimental changes at the cell and molecular level over time [19,20,21,22]. Initially, it was proposed that the resultant damage due to unscavenged ROS, which in turn can damage proteins, lipids, and DNA [15,18,23,24], may cause cellular attrition, mediating the ageing process through physiological decline, resulting in eventual death [12,13,14,22,25,26,27,28]. This hypothesis was named the oxidative stress theory of ageing (OSTA) or the free radical theory of ageing (FRTA) [12,13,14,22,25,26,27,28]. Despite the negative connotation to ROS, they are essential for normal physiological function and are involved in cellular signalling [29], inflammation response [30], altering glucose uptake and metabolism [31], and the immune response [11], allowing for the processes associated with the dealing of tolerating hypoxic stress [32], osmoprotective signalling [33], and the control of ventilation, nerve transmission, and immune regulatory processes [34]. However, it is crucial to consider that not all ROS can be scavenged and that some damage will always transpire [18,22,28,35,36]. In general, oxidative stress and the resulting damage can be due to a variety of factors, but it is primarily due to either the increased production of pro-oxidants (compounds that induce oxidative stress, either by generating reactive oxygen species or by inhibiting antioxidant systems), diminished antioxidant levels, depletion of essential dietary metal cofactors which potentiate the activity of antioxidant enzymes, and/or a failure in the repair of replacement systems [4]. It is thus generally accepted that mild elevations of ROS can be beneficial through hormesis {an adaptive response of cells and organisms to moderate (usually intermittent) stress} [16,32,37,38], where very high ROS levels are considered detrimental [20,21,39,40].

The OSTA generally shows age is positively correlated with oxidative damage [14,41,42], though some species, even when size is considered [41,43,44], contradict this correlation [20,25,26,45]. These exceptions include primates [46,47], birds [48,49,50], bats [51,52,53,54], as well as mole-rats [51,55,56,57,58,59,60,61]. These species are longer lived than others, likely through mechanisms that may mitigate the oxidative damage-induced changes usually associated with ageing [26,36,51,62] that affect the maximum lifespan potential (MLSP) [41,63,64]. The most well-known species to violate the OSTA is the naked mole-rat (*Heterocephalus glaber*) (NMR) [36,45,57,62,64,65,66,67]. Research on ageing in the NMR has demonstrated that they, even at a young age, exhibit extremely high levels of oxidative damage with no detriment to longevity, while the only finding of an oxidative damage limitation involved the insignificant increase across age [57,65]. Some factors have been identified which contribute to the longevity of the NMR, such as the poly-unsaturated fatty acid (PUFA) composition relative to saturated fatty acids to similarly aged mice [67,68], calorie-restriction-like symptoms such as a reduced metabolic rate, reduced body temperature, and reduced food consumption [69,70], and low circulating levels of methionine [69]. It is believed the main reason NMRs possess such unique characteristics is due to their exclusively subterranean lifestyle [71,72,73]. Despite the interest in NMRs as a conundrum in the oxidative stress theory of ageing [36,45,57,62,64,65,66,67], a comparison of their redox status with that of other Bathyergidae has not previously been carried out. This is surprising, as oxidative markers in tissues [45] and plasma [74] were previously found to differ, and Dammann [51] proposed that the importance of oxidative stress in bathyergids may be underestimated if only NMRs and mice are compared, or that the impact of oxidative stress may very well differ between the NMR and other African mole-rat species. Comparisons between the NMR and other African mole-rat species may allow for identifying the ecological and physiological factors involved in redox biology that are not apparent when considering only one species. Additionally, some other mole-rats, like the Damaraland mole-rat (*Fukomys damarensis*) (DMR), share multiple characteristics in common with the NMR but show a higher degree of similarity with humans in some aspects of their physiology, where this higher degree of similarity of the DMR to humans, in contrast to the NMR, may offer alternative insights to human biomedical research [75]. This may also be applicable to other African mole-rats and not just the NMR and the DMR.

Therefore, this study sought to investigate the comparison of the redox status between family members of Bathyergidae due to their unique physiology, life-history, and reproductive system as well as the current understanding of their oxidative ecology (Supplementary File S1) [22,51,55,71,74,76,77,78,79,80,81,82,83,84,85,86,87,88,89,90,91,92,93,94,95,96,97,98,99,100,101,102,103,104,105,106,107,108,109,110,111,112,113]. The current study included eight different subspecies and species of African mole-rats, namely the NMR, the DMR, the highveld mole-rat (*Cryptomys hottentotus pretoriae*) (CHP), the common mole-rat (*C. h. hottentotus*) (CHH), the Natal mole-rat (*C. h. natalensis*) (CHN), the Cape mole-rat *(Georychus capensis*) (GC), and the Cape Dune mole-rat (*Bathyergus suillus*) (BS) [77,80,114] (Table 1). These representatives were chosen due to their available data for the oxidative markers investigated, as well as representing some eusocial [96,97], social, and solitary species that vary in their resting metabolic rate (RMR) [76,115] (Table 1).

The main objective of this study was to establish how NMRs are different from, or similar to, other species from the family Bathyergidae with regards to their plasma oxidative markers, namely, total antioxidant capacity (TAC), total antioxidant status (TOS), and the ratio between these two (TOS:TAC) variables as an arbitrary measure of an oxidative stress index (OSI), and how these oxidative markers may vary with maximum lifespan potential (MLSP), RMR, the aridity index (AI), and sociality. These relationships were analysed through a phylogenetically controlled comparison of all captive mole-rat species (Table 1), wild-caught and captive comparisons (CHP and CHH), as well as wild comparisons of subspecies (CHN, CHH, and CHP) from habitats differing in their AI to determine the possible influences of the environment on oxidative markers. We also opted for a principal component analysis (PCA) to avoid analysing oxidative markers in a vacuum and determine their relationship simultaneously with variables of interest (MLSP, AI, RMR, and sociality). In order to make the data standardised, we opted to use captive individuals for the phylogenetic relationship and PCA analyses to avoid the complications of ecology (environmental factors) on plasma oxidative markers. We further opted to utilise the same markers analysed from the same laboratory, as markers measured from different laboratories are rarely comparable [57]. Also, due to the confounding effects known in social mole-rats species, namely a reproductive division of labour [74,79], we used only the non-breeding colony members of social mole-rats and solitary mole-rats individuals outside their breeding season for the current study. We realise that the markers investigated do not include DNA damage, an important aspect associated with ageing [24,28,122]; however, this study was undertaken as an initial overview of oxidative stress and ageing in this unique rodent family.

These analyses allow us to investigate: (1) Is there a phylogenetic relationship of plasma markers between African mole-rats? (2) To what extent does sociality explain the differences in oxidative markers? (3) Do oxidative markers correlate with the MLSP of African mole-rats? (4) Do oxidative markers change between captive and wild-caught species? And (5) Does the aridity of the environment influence oxidative markers?

## 2. Materials and Methods

### 2.1. Ethics Statement

The University of Pretoria, Faculty of Veterinary Science Animal Ethics Committee, approved all experimental animal procedures under the NAS 068/2021 and NAS209/2021 project codes. In addition, DLRDD section 20 approval (SDAH-Epi-12/11/1/4/1 (1948 LH) and SDAH-Epi-21031811071) was also obtained along with relevant provincial animal capture permits (Western Cape: CN44-87-13780; Northern Cape: FAUNA 0419/2021, FAUNA 042/2021; Gauteng: CPF6-0124). All methods were performed following the relevant guidelines and regulations. In addition, all experimental procedures were carried out under the recommendations in the Guide for the Care and Use of Laboratory Animals of the National Institutes of Health [123].

### 2.2. Novel Data Analysis

#### 2.2.1. Solitary Mole-Rats

Solitary mole-rats (GC and BS) were wild-caught during the non-breeding season (September–October) in 2021 close to the town of Darling (33°22′ S 15°25′ E) in the Southwestern Cape, South Africa. Animals were caught using Hickman live traps baited with sweet potatoes [124]. Once captured, the animals were transported to the mole-rat laboratory at the Department of Zoology and Entomology (25°45′13.3″ S, 28°13′50.9″ E), University of Pretoria, Hatfield, South Africa. These solitary species were kept in captivity for more than six months prior to sample collection (Table 1).

#### 2.2.2. Social Mole-Rats

Wild-caught CHH used in the current study were captured at Klawer (31.7730° S, 18.6247° E) during the non-breeding season between November 2021 and March 2022. The social mole-rats were captured and transported as outlined for solitary mole-rats. A subset of animals (Table 1) was sampled within 72 h of being in captivity (similar to other studies [79,94]). An additional subset was maintained under laboratory conditions for more than six months before sampling.

All animals used in this study were considered adults and reproductively inactive (non-breeders) {see Bennett and Faulkes [125] on how reproductive status was determined} (Table 1). The use of non-breeders avoids complications between breeder and non-breeder comparisons associated with oxidative stress due to reproduction [74,79] and the effects of reproductive suppression differences [74,79,98,99,100,101,102,103,104,105,106,125,126] (Supplementary File S1).

#### 2.2.3. Mole-Rat Blood Sample Collection

All plasma was collected between 08h00 and 13h00 to prevent the circadian rhythmicity of oxidative markers [127,128]. The body mass of each animal was recorded to the nearest 0.01 g (Scout Pro SPU123; Ohaus Corporation, Pine Brook, NJ, USA) (Table 1). All mole-rats were handheld, and venous blood samples were collected from the hindfoot, tail, or heart, if euthanised with an overdose of isoflurane. Approximately 300–500 µL of blood was collected from each animal. All blood was centrifuged at 13,300 rpm, and the resulting plasma was decanted and stored at −70–80 °C until further analysis.

#### 2.2.4. Oxidative Stress Markers

The oxidative stress markers of TOS, TAC, and OSI were measured in captive and wild CHH, as well as captive CHP, captive GC, and BS (Table 1).

##### Reagents

Unless otherwise stated, all chemicals and reagents used in this study were obtained from Merck (Pty) Ltd. (Gauteng, South Africa).

##### Total Antioxidant Capacity (TAC) Assay

Plasma TAC levels were quantified using a commercially available kit (Antioxidant Assay Kit, Cayman Chemical Co., Ann Arbor, MI, USA) which measures the oxidation of ABTS (2,29-Azino-di-[3-ethybenzthiazoline sulphonate]) by metmyoglobin, which is inhibited by the non-enzymatic antioxidants contained in the sample. Oxidised ABTS is measured by spectrophotometry at a wavelength of 750 nm. The capacity of antioxidants in the sample to inhibit the oxidation of ABTS is compared with the capacity of known concentrations of Trolox, and the results are expressed as micromole Trolox equivalents per litre (μmol Trolox equivalents/L). Samples were run in duplicate and only once per plate with a repeatability of r = 0.96. The intra-assay variability (%CV) was 3.65%.

##### Total Oxidant Status (TOS) Assay

Plasma TOS levels were measured with Erel’s method [129]. Briefly, this method is based on the oxidation of ferrous ions to ferric ions in the presence of various oxidative species. The oxidation reaction is enhanced by glycerol molecules, which are abundantly present in the reaction medium. In an acidic medium, the ferric ion makes a coloured complex with xylenol orange. The colour intensity, measured using a spectrophotometer, is related to the total amount of oxidant molecules that are present in the sample. The results are expressed in terms of micromole hydrogen peroxide equivalent per litre (μmol H_2_O_2_ equivalent/L). Samples were run in duplicate and not repeated once per plate with a repeatability of r = 0.99. The intra-assay variability (%CV) was 4.5%.

##### Oxidative Stress Index (OSI)

Oxidative stress was determined by the TOS:TAC ratio, which represents the oxidative stress index (OSI) arbitrary unit, which was calculated as follows: OSI = [(TOS, μmol H_2_O_2_ equivalent/L)/(TAC, mmol Trolox equivalent/L)].

### 2.3. Multi-Species Analyses

#### 2.3.1. Animal Housing

All animals were housed in the mole-rat laboratory of the Department of Zoology and Entomology at the University of Pretoria, South Africa, and all animals were maintained under similar conditions. Animals were housed in climate-controlled rooms within their thermal neutral zone (TNZ) [76] and were maintained on a 12L:12D photoperiod with 50–60% relative humidity. The mole-rats were fed daily on sweet potatoes and apples ad libitum. All animals were fed the same variety of chopped vegetables and drank no free water.

#### 2.3.2. Resting Metabolic Rate of Mole-Rats

The mass-specific resting metabolic rates (msRMR) for each mole-rat species for wild and captive mole-rats were obtained through several studies (Table 1). These msRMR values for a species were multiplied by the individual’s body mass to estimate each individual RMR, hereafter referred to as ERMR.

### 2.4. Aridity Index Data

The AI was gathered using the methods outlined by Jacobs et al. [94]. Climate data for each species were retrieved from ERA5-Land of the European Centre for Medium-Range Weather Forecasts—the latest generation created by the Copernicus Climate Change Service [130]. The spatial resolution is 0.1 × by 0.1. These data were used to calculate an annual *AI* (Equation (1)). Whereas the total precipitation (*tp*) was directly obtained from ERA5-Land, the potential evapotranspiration (*PET*) was calculated from the well-known Romanenko estimation (Equation (2)) [131]. For Equation (2), the relative humidity (*RH*) was calculated from ERA5-Land d2m (Equation (3)).

(1) $$AI=\frac{tp}{PET}$$

(2) $$PET\;0.0006\;\times \;\left(100-RH\right)\times \;{\left(25+Tair\right)}^{2}$$

(3) $$RH=100\;\times \;10^{7.591386(\frac{d2m}{d2m+240.7263}-\frac{Tair}{Tair+240.7263})}$$

### 2.5. Phylogenetic Tree Determination

**Figure 1:**
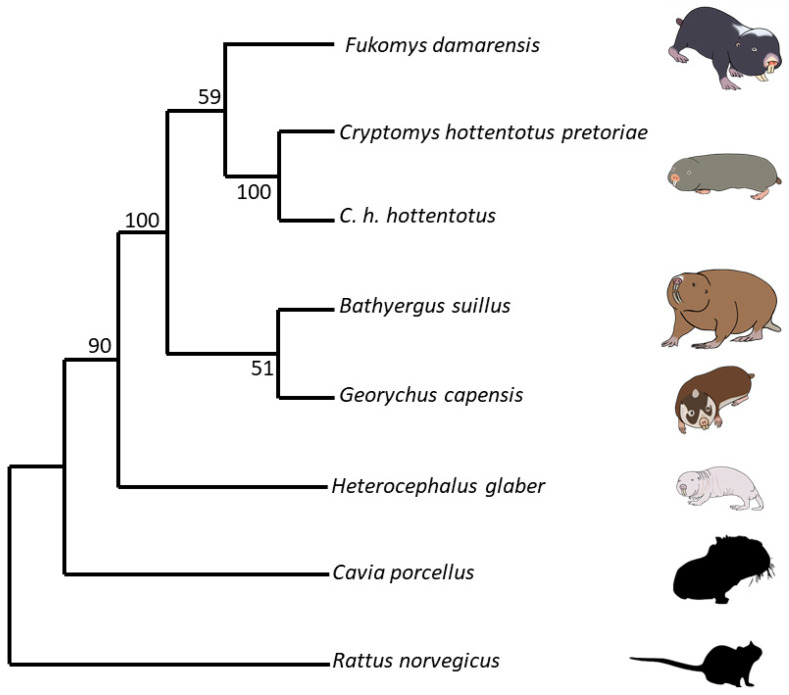
The optimal phylogenetic tree of five different captive mole-rat species and two subspecies, the naked mole-rat, *Heterocephalus glaber*, the Damaraland mole-rat, *Fukomys damarensis*, the highveld mole-rat, *Cryptomys hottentotus pretoriae*, the common mole-rat, *C. h. hottentotus*, the Cape mole-rat, *Georychus capensis*, and the Cape Dune mole-rat, *Bathyergus suillus*, as well as two outgroup species, the brown rat, *Rattus norvegicus* and the Guinea pig, *Cavia porcellus*. The tree is drawn to scale, with branch lengths being the same units as those of the evolutionary distances used to infer the phylogenetic tree. The evolutionary history was inferred using the Neighbour-Joining method [142]. The percentage of replicate trees in which the associated taxa clustered together in the bootstrap test (1000 replicates) are shown next to the branches [143]. Evolutionary distances were computed using the Maximum Composite Likelihood method [144] and are in the units of the number of base substitutions per site. This analysis involved eight nucleotide sequences. All ambiguous positions were removed for each sequence pair (pairwise deletion option). There were a total of 1201 positions in the final dataset.

Cytochrome b gene sequence fasta files for each species were obtained from the existing GenBank data [132]. This involved the cytochrome b sequence for mole-rats, namely, *Heterocephalus glaber* Accession No. MT8453841 [133], *Fukomys damarensis* Accession No. AF012223.1 [134], *Cryptomys hottentotus pretoriae* Accession No. AF012236.1 [134], *Cryptomys hottentotus hottentotus* Accession No. MH186559.1 [135], *Bathyergus suillus* Accession No. KJ866687.1 [136], and *Georychus capensis* Accession No. U18837.1 [137]. We also obtained the cytochrome b sequences for the two outgroups, namely, *Rattus norvegicus* Accession No. KT024821.1 [138] and the Guinea pig *Cavia porcellus* Accession No. AY382793.1 [139]. Aligning was carried out using Mesquite version 2.75 [140]. Tree building was carried out using the Molecular Evolutionary Genetic Analysis Version 11 (MEGA11) program [141]. Mesquite files were converted to mega files and, using the “Phylogeny” menu, neighbour-joining trees were created using the Neighbour-Joining method [142]. The percentage of replicate trees in which the associated taxa clustered together in the bootstrap test (1000 replicates) are shown next to the branches [143]. The evolutionary history was inferred using the evolutionary distances computed using the Maximum Composite Likelihood method [144] and are in the units of the number of base substitutions per site. This analysis involved eight nucleotide sequences. The best DNA substitution model for each set of sequence alignments was found using the “Models” menu, and this was used to create the trees. All ambiguous positions were removed for each sequence pair (pairwise deletion option). There were a total of 1201 positions in the final dataset. Evolutionary analyses were conducted in MEGA11 [145]. The optimal tree is shown and drawn to scale, with the branch lengths the same units as those of evolutionary distances used to infer the phylogenetic tree (Figure 1).

### 2.6. Statistical Analysis

Unless specified, all calculations, statistical, and visual analyses were performed using the statistical software R version 4.2.2 [146] and GraphPad Prism 8.4.3. Data are presented as the mean ± standard error (s.e.m), and a *p*-value of ≤0.05 indicates significance. The normality of the response variables TOS, TAC, and OSI, was determined using Shapiro–Wilk tests, and the homogeneity of all dependent variables was checked with Levene’s test.

#### 2.6.1. Phylogenetic Analysis of Oxidative Variables

Individuals from five mole-rat species, of which two in the genus *Cryptomys* were two subspecies, (NMR, DMR, CHH, CHP, GC, and BS) that had been housed in captivity for longer than six months were used in the phylogenetic analysis. A generalised variance inflation factor (GVIF) was used to determine multicollinearity between the life history traits to account for the mix of continuous and categorical traits, undertaken in a stepwise fashion. A generalised linear mixed-effects model using a Markov chain Monte Carlo approach under a Bayesian statistical framework (MCMCglmm) was applied in the ‘MCMCglmm’ package [147]. This methodology was used to incorporate the multiple studies per subspecies or species that were present in the database. This approach fits the individual-level data whilst controlling for relationships in species traits due to common ancestry. A single consensus tree was used, and 130,000 iterations were applied with 100 thinning intervals and 30,000 burn-in. ERMR, AI, and sociality were used as predictor variables. The statistical significance of the genetic influence on TOS/TAC/OSI was assessed using 95% confidence intervals (CI) for the heritability estimates, which is the transmission of the phenotypic variability within a population from generation to generation, and a heritability value (H^2^) was obtained. All calculations and statistical analyses were performed using the statistical software R version 4.2.2 [146].

#### 2.6.2. Wild-Caught and Captive Mole-Rats Comparison

Wild-caught and captive mole-rat comparisons between the CHH and CHP used oxidative markers (TAC, TOS, and OSI) as the response variable and used the subspecies and status (wild-caught/captive) and their interaction as predictors, with ERMR as a covariate. All values violated the assumptions of normality and homoscedasticity; and as such, we used a generalised linear model (GZLM) with a gamma distribution with an inverse link function using the *lme4* package [148]. Models were model selected using the MuMIn package [149] and dredged with a delta function <2. If only one model was given, then that model was accepted as the best model. Significant variables in the linear modes were followed up with post-hoc comparisons, conducted using Tukey’s HSD pairwise comparisons using the *emmeans* package [150]. Only relevant biological comparisons were presented in the results.

#### 2.6.3. Wild-Caught Mole-Rat Comparison

For the analyses between the wild CHP, CHH, and CHN, we used subspecies as our predictor and ERMR as a covariate. This analysis was used to infer whether species differences exist due to these species occupying different AI habitats (Table 1). All values violated the assumptions of normality and homoscedasticity; as such, we used a GZLM with a gamma distribution with an inverse link function using the *lme4* package [148]. Models were model selected using the MuMIn package [149] and dredged with a delta function <2. If only one model was given, then that model was accepted as the best model. Significant variables in the linear modes were followed up with post-hoc comparisons, conducted using Tukey’s HSD pairwise comparisons using the *emmeans* package [150]. To prevent pseudo-replication when determining the aridity influence on wild-caught mole-rats’ oxidative variables, the means of TAC, TOS, and OSI of each species were correlated using simple linear regressions with an AI from where the animals were caught.

#### 2.6.4. Principal Component Analysis (PCA)

We opted to perform a PCA analysis due to the limitations of our variables being multicollinear. It has been proposed that PCAs can help investigate oxidative balance [151]. Briefly, this analysis allows for identifying the dimensionality of the oxidative variables within a context, with their loadings inferring how they interact within this dimensionality [151]. Furthermore, the axes, such as principal components (PCs), group several variables together, and that can be analysed as a new variable that can better represent the underlying process involved, compared to analysing the individual oxidative markers themselves [151]. Our study used the three oxidative markers (TAC, TOS, and OSI), AI, ERMR, and body mass. Furthermore, we also opted to use the MLSP of each species as determined through AnAge open-source dataset [121] and the mole-rat laboratory longevity data (Table 1) as our seventh component, which was applied to the six captive mole-rat species. The PCA was performed using the *prcomp* function using the ggplot2 package [152]. The number of PCs was determined through Eigenvalues, *skree* plots, and the cumulative proportion of variance explained by the PCs [153]. We used the Kaiser criterion to determine the number of PCs to analyse [153]. The functions *ggbiplot* from the ggplot2 package [152] and *cor* were used to determine the relationship of the variables within each component and the loading of the variables within each PC was visualised as a biplot [154]. The ellipse probability was set to 68%.

## 3. Results

### 3.1. Phylogenetic Analysis of TOS, TAC, and OSI

**Figure 2:**
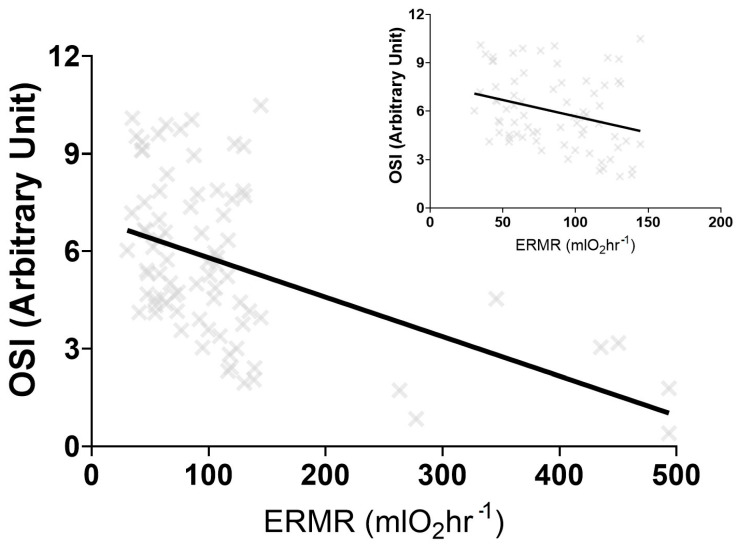
The significant negative linear regression output between the oxidative stress index (OSI) and the estimated resting metabolic rate (ERMR) for five different captive mole-rat species and two subspecies, the naked mole-rat, *Heterocephalus glaber*, the Damaraland mole-rat, *Fukomys damarensis*, the highveld mole-rat, *Cryptomys hottentotus pretoriae*, the common mole-rat, *C. h. hottentotus*, the Cape mole-rat, *Georychus capensis* and the Cape Dune mole-rat, *Bathyergus suillus*. The negative relationship remains significant when *B. suillus* is removed (figure insert).

Phylogenetic heritability (*H*^2^) or the lambda (λ) for TOS, TAC, and OSI suggest phylogeny does not strongly predict oxidative stress variables. Sociality and AI were not significant predictors for TOS, TAC, and OSI (Table 2). Similarly, ERMR was not a significant predictor for TOS and TAC but was a significant predictor of OSI (Table 2). A linear regression analysis between OSI and ERMR demonstrated a significant negative correlation (R^2^ = 0.2301, F(_1,78_) = 23.23, *p* < 0.001) (Figure 2). This linear regression remained significant when the BS was removed (R^2^ = 0.09, F(_1,71_) = 6.844, *p* = 0.011) (Figure 2).

### 3.2. Wild-Caught and Captive Mole-Rats Comparison

#### 3.2.1. TOS

**Figure 3:**
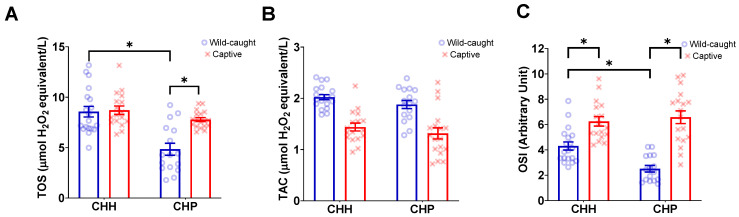
The plasma oxidative markers, (**A**) total oxidant status (TOS), (**B**) total antioxidant capacity (TAC), and (**C**) oxidative stress index (OSI) of wild-caught common mole-rats, *Cryptomys hottentotus hottentotus* and highveld mole-rats, *C. h. pretoriae* (blue bars and empty circles) compared to captive common mole-rats and highveld mole-rats (red bars and crosses). Bars represent the mean ± s.e.m. An asterisk (*) indicates significance (*p* ≤ 0.05).

The best model did not retain ERMR for wild-caught and captive mole-rats (CHP and CHH) (Table 3). At the same time, species and captivity did not affect TOS (Table 3). The interaction between subspecies and status (wild-caught or captive) was significant (Table 3, Figure 3A). Post-hoc analyses revealed that wild-caught CHP had a significantly lower TOS compared to captive CHP (t = −4.434, *p* = 0.0002, Figure 3A). Additionally, wild-caught CHP had a significantly lower TOS as compared to wild-caught CHH (t = −5.254, *p* < 0.0001, Figure 3A). No significant differences were observed between the TOS of captive and wild-caught CHH (t = −0.175, *p* = 0.9981, Figure 3A).

#### 3.2.2. TAC

The best model did not retain ERMR for wild-caught and captive mole-rats (CHP and CHH) (Table 3). Only status (wild-caught vs. captive) was significant (Table 3), where wild-caught individuals had a higher TAC (1.96 ± 0.04 mmol Trolox equivalents/L) compared to captive individuals (1.37 ± 0.07 mmol Trolox equivalents/L) (Figure 3B). Subspecies or species and the interaction between these and status did not significantly influence TAC (Table 3; Figure 3B).

#### 3.2.3. OSI

The ERMR for wild-caught and captive mole-rats (CHP and CHH) was not retained in the best model (Table 3). The best model demonstrated the significance of the main effect of status, where the wild-caught OSI was lower (3.49 ± 0.25) compared to captive mole-rats (6.42 ± 0.31), and the interaction between species and status was significant (Table 3, Figure 3C). Post-hoc analysis revealed that wild-caught CHP had a significantly lower OSI compared to captive CHP (t = −7.088, *p* < 0.0001, Figure 3C). Furthermore, wild-caught CHP had a significantly lower OSI than wild-caught CHH individuals (t = −4.472, *p* = 0.0002, Figure 3C). Interestingly, wild-caught and captive CHH significantly differed in their OSI, whereas wild-caught CHH had a significantly lower OSI compared to captive CHH (t = −3.344, *p* = 0.0073, Figure 3C).

### 3.3. Wild-Caught Mole-Rat Comparison

#### 3.3.1. TOS

**Figure 4:**
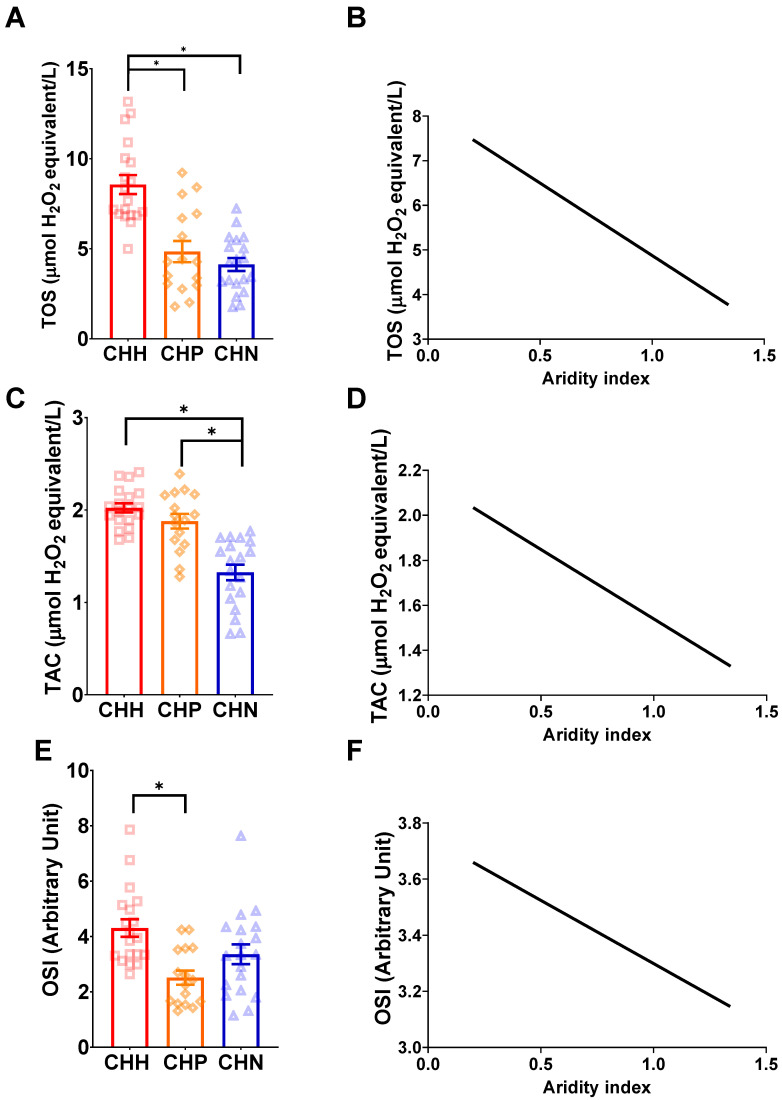
The relationship between oxidative markers and the aridity index between three different wild-caught mole-rats, namely common mole-rats, *Cryptomys hottentotus hottentotus* (CHH—red with squares), highveld mole-rats, *C. h. pretoriae* (CHP—orange with diamonds) and the Natal mole-rat, *C. h. natalensis* (CHN—blue with triangles) for (**A**,**B**) total oxidant status (TOS), (**C**,**D**) total antioxidant activity (TAC), and (**E**,**F**) oxidative stress index (OSI). Bars represent the mean ± s.e.m. An asterisk (*) indicates significance (*p* ≤ 0.05).

The ERMR for wild-caught mole-rats (CHP, CHH, and CHN) was not retained in the best model (Table 4). A significant effect of species was observed in the model (Table 4). Post-hoc comparisons between the three subspecies demonstrated that CHH had significantly higher TOS as compared to CHN (t = −5.328, df = 51, *p* < 0.0001) and CHH had a significantly higher TOS than CHP (t = −4.040, df = 51, *p* = 0.0005), but CHN and CHP did not significantly differ in TOS (t = 1.236, df = 51, *p* = 0.44) (Figure 4A). Simple linear regressions revealed that the TOS has a significant negative relationship with an increasing aridity index (R^2^ = 0.3221, F(_1,52_) = 24.7, *p* < 0.0001) (Figure 4B).

#### 3.3.2. TAC

The best model did not retain the ERMR for wild-caught mole-rats (CHP, CHH, and CHN) (Table 4). A significant effect of subspecies was observed in the model (Table 4), where post-hoc comparisons between the three subspecies demonstrated that CHN had a significantly lower TAC compared to CHH (t = −6.319, df = 51, *p* < 0.0001) and CHP (t = 5.122, df = 51, *p* < 0.0001) (Figure 4C). We also found that CHH had a higher TAC than CHP, but this was not significant (t = −1.083, df = 51, *p* = 0.53) (Figure 4C). Simple linear regressions revealed that TAC had a significant negative relationship with an increasing aridity index (R^2^ = 0.5145, F(_1,52_)= 55.10, *p* < 0.0001) (Figure 4D).

#### 3.3.3. OSI

The ERMR for wild-caught mole-rats (CHP, CHH, and CHN) was not retained in the best model and was found to be significant (Table 4). A significant effect of subspecies was observed in the model (Table 4). Post-hoc comparisons between the three subspecies demonstrated that CHH had a significantly higher OSI as compared to CHP (t = −3.657, df = 51, *p* = 0.002) and higher OSI as compared to CHN, but this was not significant (t = −1.890, df = 51, *p* = 0.15) (Figure 4E). The OSI did not significantly differ between CHN and CHP (t = −2.061, df = 51, *p* = 0.11), whereas the CHN had a slightly higher OSI (Figure 4E). A simple linear regression found a non-significant relationship between the OSI and the aridity index (R^2^ = 0.022, F(_1,52_)= 1.174, *p* = 0.28) (Figure 4F).

### 3.4. Principal Component Analyses of Species

PCA plots revealed underlying factors in the plasma oxidative markers for species. Since sociality is not a continuous variable, species demarcation allows for the influence of eusociality, sociality, and solitary lifestyles for interpretation. The benefit of using a PCA over the previous analyses is the high likelihood of multicollinearity among variables, thus avoiding that complication and allowing for a better understanding of the relationship of these variables to each other.

**Figure 5:**
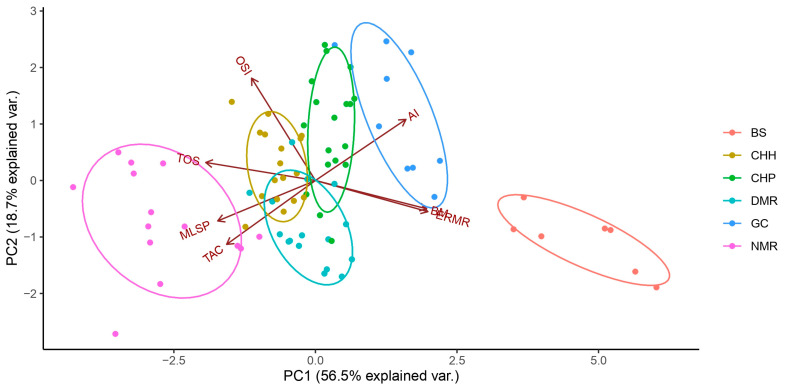
Principal component analysis illustrating the relationships between the total oxidant status (TOS-µmol H_2_O_2_ equivalents/L), total antioxidant capacity (TAC-mmol Trolox equivalents/L), oxidative stress index (OSI-arbitrary unit), maximum lifespan potential (MLSP), aridity index (AI), body mass (BM), and estimated resting metabolic rate (ERMR) for captive mole-rat species, namely, the naked mole-rat *Heterocephalus glaber* (NMR), the Damaraland mole-rat, *Fukomys damarensis* (DMR), the highveld mole-rat, *Cryptomys hottentotus pretoriae* (CHP), the common mole-rat, *C. h. hottentotus* (CHH), the Cape mole-rat, *Georychus capensis* (GC), and the Cape Dune mole-rat, *Bathyergus suillus* (BS). PC1 explained 55% of the variance and PC2 explained 20.3% of the variance for a cumulative variance of 75.4%. The direction of the arrows represents the loadings of the variables within a PC.

Using the skreeplot, we identified two PCs with an Eigenvalue above one; as such, we utilised two PCs (PC1 and PC2). PC1 and PC2 cumulatively explained 75.3% of the variance (Figure 5). The loading of these variables within each PC was determined using linear correlations and visually represented in the direction of the arrows (Figure 5).

PC1 separated the species and subspecies mainly in two directions, where species with a higher body mass and ERMR were found in habitats with a higher aridity index (Figure 5). However, the direction of those three variables (BM, ERMR, and AI) negatively influenced TOS, TAC, OSI, and MLSP (Figure 5). This suggests that the more social species and subspecies (NMR, DMR, CHP, and CHH) orientated more to the left have a higher TOS, TAC, and OSI, but interestingly, would tend to have a higher MLSP (Figure 5). Contrastingly, the solitary species (GS and BS) are found on the right of the PCA, suggesting a lower TAC, TOS, and OSI, and tend to have a lower MLSP (Figure 5). PC2 separated species primarily on OSI, TAC, and the aridity index, implying that species and subspecies typically have a higher OSI and lower TAC as the aridity index increases (Figure 5). The other variables explained the minimal variance to these three factors (correlation < 0.25).

## 4. Discussion

This study investigated several factors which could have a possible influence on oxidative marker levels, such as phylogeny [155,156], sociality [72,157,158], wild-caught and captive differences [159], aridity [117], RMR [40,43,160], and MLSP [41,63,64]. Body mass and RMR have previously been shown to correlate with free radical production, where a higher metabolic rate would assume increased free radical production [12,160,161], with some exceptions [43,162,163]. Oxidative stress in species with increased MLSP is generally lower, whereby they either change the rate of production or have additional means of protection [13,41,164]. African mole-rats present a conundrum as they typically have lower metabolic rates and high oxidative stress, yet they are also long-lived. Our goal in this study was to identify, from the data available, how some oxidative markers correlate within and between subspecies and species of African mole-rats, where markers can be directly compared. Our current analyses provide insights into the critical processes that may influence the oxidative markers in African mole-rats. These observations include: (1) oxidative markers and sociality may explain some patterns associated with longevity but are likely outweighed by other factors, (2) a positive association between oxidative stress and MLSP, and (3) oxidative markers can vary due to changes in RMR and aridity, which may be more profound in some subspecies and species compared to others. There are some limitations to the current study, with the most notable including the limited number of species and subspecies of Bathyergidae investigated in this study. Some excluded species include the highly social giant mole-rat, *F. mechowii*, and some solitary mole-rats, namely the silvery mole-rat, *Heliophobius argenteocinereus*, and Namaqua Dune mole-rat, *B. janetta*. Namaqua Dune mole-rat data are hard to obtain due to species decline in the arid habitat (N.C. Bennett and D.W. Hart personal communication), resulting in their exclusion from this analysis. Additional shortcomings include the lack of additional oxidative markers, such as DNA-damage markers and enzymatic antioxidants.

Sociality and longevity are key aspects of the life history of a species [158,165,166], i.e., Williams and Shattuck [157] identified that eusociality and habitat play a prominent role in determining longevity. Zhu et al. [167] also provided further evidence for social subspecies and species having greater longevity in mammalian phylogenies as compared to solitary species. Previously, sociality has been correlated to longevity in insects [168]. It was proposed that African mole-rats’ subterranean and social lifestyle contribute to their longevity [72,157], but Healy [169] proposed that being subterranean is not associated with MLSP. Our analysis emphasises habitat (determined through the AI) and sociality (social species tend to be more to the left of the PCA analysis) to be two components determining oxidative markers and orientation towards MLSP of the PCA. According to the aridity food distribution [170] and behavioural osmoregulation hypotheses [115], animals in habitats with increased aridity tend to be more social, and our data agree with this. However, this increased aridity congruently contributes to elevated oxidative stress, which seems to be promoting longevity in the social subspecies and species. One outlier in the study is that the solitary GC appears closer to the social species as opposed to the BS. Because the GC is a solitary species, it is expected to trend away from MLSP in our PCA. Since this was not the case, oxidative markers and the increased MLSP of a species are likely closely tied to other factors and not sociality, despite sociality contributing to the observation (social species tend to be more to the left in the direction of MLSP). Furthermore, Dammann, Šaffa and Šumbera [72] have also shown extended longevity in the solitary silvery mole-rat. They also agreed that sociality promotes longevity in this family [72]. Thus, our results agree with Williams and Shattuck [157] and partially disagree with Healy [169], emphasising that sociality plays a strong role in the correlations of oxidative markers to the MLSP in the family Bathyergidae but that habitat may have a greater influence.

Increased ROS species formation has been predicted as a significant determinant of the ageing process mediated through metabolic rate-producing free radicals [12,23,40,63]. Despite the simplicity of this hypothesis, recent evidence suggests that this link is not so straightforward and even contradicting. The general trend accepts that reducing oxidative stress promotes longevity, which is closely linked to body mass, metabolic rate, and the rate of free radical production [15,41]. Astoundingly, some cases report increased ROS formation to promote health and lifespan [40]. This phenomenon may likely manifest under a biphasic response of hormesis [32,37,38,171], where it has previously been shown that hormetic effects improve survival under chemical challenges such as hypoxia [32,172], where an elevated oxidative stress profile is expected in order to upregulate defences. This highlights a fundamental question: despite the high oxidative lipid peroxides in some social African mole-rats, what is the baseline to indicate a detrimental oxidative stress level to these mole-rats? Our data support the idea that increased oxidative stress benefits animals, resulting in increased MLSP.

Previously, it has been shown that the overall levels of antioxidants among mammalian species do not correlate with MLSP [45,173]. Since we compared captive individuals, food provisioning can affect antioxidant levels, as non-enzymatic antioxidants are primarily obtained from food [174,175]. Even with an extended time in captivity, these TAC differences are still present, suggesting species differences in the inter-organ transport of antioxidants [176,177]. Previously, NMRs have been suggested to have poor antioxidant activity [178,179], where the antioxidant activity was proposed to be independent of the high MLSP observed [45,180]. Munro et al. [60] found significantly higher antioxidant defences for NMRs, as NMRs consume hydrogen peroxide at a much higher rate in the matrix of mitochondria. In our study, NMRs had the highest TAC of all captive animals, which suggests NMR have the most effective non-enzymatic antioxidant activity (Supplementary File S2). Furthermore, the TAC in our PCA analyses contributes to the MLSP of the species. Importantly, NMRs were similar to the other social mole-rats and the solitary GC when considering OSI, while only the DMR and BS had a lower OSI. Lastly, since the GC demonstrated a similar OSI to the social species, but vastly different TOS and TAC, it suggests that the GC have a similar ecological mechanism as their OSI is similar to social mole-rats and maintains a higher MLSP than BS.

Emerging evidence suggests that an extended lifespan may be maintained by natural selection as a product of organismal adaption [181], where the majority of longevity attributes are under genetic regulation [182,183], and that these genetic regulators are constrained by environmental factors [184,185]. Hypoxia tolerance is an adaptation that may be one of the most relevant pathways promoting longevity in African mole-rats [73,88,89,186]. African mole-rats live in a subterranean environment, which is hypoxic and hypercapnic [72,125,157], promoting factors such as optimising oxygen uptake or reducing the oxygen requirement for metabolism [187]. The most well-known hypoxia-driven adaptation involves selection on the central metabolism, cellular respiration, haemoglobin-mediated oxygen transport, hypoxia-inducible factor pathways, and decreased thermogenesis [181,188,189,190]. Hypoxia adaptation induces hypothermia and hypo-metabolism, which are physiologically similar to calorie-restricted animals [70,191] and linked to extended lifespan [69,71]. The adaptation of living in a subterranean habitat gave way to these attributes [71,157], which suggests that for oxidative stress, hypoxia tolerance as an adaptation to a subterranean lifestyle promotes MLSP and disagrees with Healy [169]. This is supported by a recent study where mice underwent metabolic remodelling by exposure to NMR-like living conditions of hypercapnia and hypoxia (as experienced as a fossorial animal), ultimately leading to these mice living longer [70]. From our data, all species except for BS are hypoxia tolerant, with the NMR being the most extreme hypoxia-tolerant species [88,89]. The consequences of hypoxia generally result in increased antioxidants and increased ROS production, which can overall result in higher oxidative stress initiating hormesis, thus promoting longevity [32,37,171]. This also supports why some of the more hypoxia-tolerant species in this study have higher levels of all oxidative markers except for GC. Two factors separate the GC from the other African mole-rats, despite similarities in OSI. They live in a mesic habitat and are generally larger with a lower mass-specific RMR [76,192], both characteristics that have been shown to alter observed oxidative marker measurements. Additionally, despite being hypoxia tolerant like the social African mole-rats species, the GC does differ in some of their responses to hypoxia compared to some of the social species [88,193]. Lastly, Ivy et al. [88] suggested that these mechanisms were independent of body size and sociality, suggesting environmental pressures from their environment gave rise to these traits.

Oxidative markers, particularly non-enzymatic antioxidants, are influenced by the immediate environment an animal finds itself in due to changes in food availability, food types, and their antioxidant content [117,174,175,194,195]. Our study emphasises the comparison between captive and wild-caught species, as wild-caught species had a significantly higher TAC than captive animals’ TAC. Our data likely support differences in diet under a wild context and possible arid-adapted mechanisms to obtain higher antioxidants, as both species had a similar TAC in captivity, supported by the significant negative relationship observed between AI and TAC. One additional mechanism that could affect differences observed in TAC is the differences in RMR, as elevated RMR may likely require additional antioxidants to combat the effects of elevated metabolism and other confounding effects of an increasingly arid environment such as water stress and thermal stress [92,93,94,196]. It may also be that antioxidants are not just readily available in foods in more arid environments to protect against droughts [194,197] but that animals in these more arid environments likely mobilise antioxidants more readily to deal with thermal challenges [176,177]. A similar observation was observed in the kidney tissues of CHH in differing arid environments [117]. Interestingly, this may suggest that oxidative kidney and plasma markers show similar changes in TAC, but this requires further investigation. Lastly, previous studies have observed differences in the RMR between wild-caught and captive species [88,92,120], but the current study did not observe significant differences in the RMR between wild-caught and captive mole-rats. This may likely be due to using an estimated measure of RMR which could not compensate for possible individual differences in RMR determination or that RMR differences between wild-caught and captive individuals are not large enough, as some species, such as the CHH, demonstrate a much larger increased in metabolic rate between captive and wild-caught individuals as opposed to the BS and CHP [88,92,120].

The study of the family Bathyergidae may have profound medical importance due to their cancer resistance, hypoxia-tolerance, and factors that promote longevity [59,66,75,198]. Other factors include the reliance on glycolysis instead of oxidative phosphorylation [186], which is enigmatic, as some African mole-rats demonstrate high oxidative stress despite the reduced ROS production from glycolysis instead of oxidative phosphorylation [199]. This highlights several vital factors for future research. Firstly, the determination of how other oxidative markers correlate, which can include other damage markers, in particular DNA, due to the relevance of DNA damage to ageing [200,201,202,203], and other antioxidant measures such as enzymatic activity [60,204] and other unconventional antioxidants such as uric acid [65,205,206] or melatonin [204,207]. Secondly, the rate of free radical production, instead of reliance on the measure of metabolism, as the metabolic rate may obscure the rate of free radical production [43]. Despite this, cellular ROS production does not correlate with longevity [208], although some studies have shown that where free radicals are produced is essential (near DNA) [35,164]. Thirdly, the susceptibility to oxidative stress and/or damage may include factors such as repair mechanisms [35,71], lipid membrane composition, which has previously also correlated to longevity [68,180], and protein structure and stability [64,209]. Lastly, excluding the domestic rat, there was a lack of plasma TOS and TAC oxidative markers available for phylogenetic-controlled redox comparisons outside the family Bathyergidae. The guinea pig had a single study [210] and there have been no studies on the African porcupine (*Hystrix africaeaustralis*), typical outgroup species used for mole-rat phylogenetic analyses [89,134]. Acquiring oxidative data from outgroup species is important to determine whether phylogeny is critical in redox physiology. As suggested, several avenues remain to be explored, not just how these factors may mediate oxidative markers but how they interact, to determine the mechanisms promoting longevity in species.

Wong, Freeman and Zhang [75] have previously stated that, despite the convergent evolution of the NMR and DMR, the DMR has demonstrated similar mechanisms to the NMR but has attracted less attention. Additionally, many physiological mechanisms for different adaptations in the NMR remain unexplored for the DMR [75]. From an oxidative stress perspective, our results show that the DMR is similar to the other social African mole-rat species, such as the *Cryptomys* sp. and GC, to some extent. In summary, we found a positive oxidative stress relationship with MLSP and a negative relationship between oxidative stress and RMR. This may be an evolutionary artefact present in African mole-rats, but we encourage further research to determine the similarities and differences of the NMR, not just to the DMR, but to the whole family of Bathyergidae.

## Figures and Tables

**Table 1:** Common names and characteristics of the seven mole-rat subspecies and species used in this study.

Subspecies and Species Name	Sociality	Aridity Index (Habitat Type)	Maximum Lifespan Potential (Years)	Body Mass (g)	Sample Size	Mass-Specific Resting Metabolic Rate (mL O_2_ h^−1^g^−1^)	Oxidative Stress Data Source
*Fukomys damarensis*	Eusocial	0.10 (Arid) [76,115]	15.5	131 ± 26	15	0.87 [88]	[74]
*Heterocephalus glaber*	Eusocial	0.08 (Arid) [76]	31	46 ± 10	14	1 [116]	[74]
*Cryptomys hottentotus pretoriae*	Social	0.48 (Semi-arid) [76,115]	11	Wild-caught 108 ± 24	16	0.82 [88]	[79]
				Captive 133 ± 32	20	0.72 [92]	This study
*Cryptomys hottentotus hottentotus*	Social	0.19 (Arid) [76,115,117]	11	Wild-caught 53 ± 16	19	1.32 [88]	This study
				Captive 68 ± 20	18	0.92 [118]	This study
*Cryptomys hottentotus natalensis*	Social	1.33 (Hyper-mesic) [100]	11	103 ± 39	19	0.79 [88]	[94]
*Georychus capensis*	Solitary	0.51 (Mesic) [76,115]	11	209 ± 33	9	0.59 [119]	This study
*Bathyergus suillus*	Solitary	0.51 (Mesic) [76,115]	6	838 ± 209	7	0.47 [120]	This study

Data represent the mean ± SD. Maximum lifespan potential data obtained from AnAge [121] or laboratory husbandry information.

**Table 2:** The best generalised linear mixed-effects models using a Markov chain Monte Carlo approach under a Bayesian statistical framework output for plasma oxidative markers, namely, the total oxidant status (TOS), total antioxidant activity (TAC), and oxidative stress index (OSI) for the five different captive mole-rat species and two subspecies, the naked mole-rat, *Heterocephalus glaber*, the Damaraland mole-rat, *Fukomys damarensis*, the highveld mole-rat, *Cryptomys hottentotus pretoriae*, the common mole-rat, *C. h. hottentotus*, the Cape mole-rat, *Georychus capensis*, and the Cape Dune mole-rat, *Bathyergus suillus* in response to sociality (eusocial:social:solitary), the aridity index (AI), and their estimated resting metabolic rate (ERMR).

	TOS		TAC		OSI	
*H*^2^/λ	0.72		0.46		0.33	
	post.mean	*p*	post.mean	*p*	post.mean	*p*
Social~Solitary	0.123572	0.912	0.582482	0.350	−0.301008	0.864
Social~Eusocial	−3.118303	0.640	0.523573	0.272	−0.573683	0.776
Solitary~Eusocial	2.615357	0.516	−0.0039322	0.892	−0.029933	0.936
AI	−1.063284	0.830	−0.435481	0.684	2.632716	0.470
ERMR	−0.009047	0.210	−0.000211	0.838	−0.012625	0.038 *

An * signifies significance.

**Table 3:** The best generalised linear model output for plasma oxidative markers, namely, the total oxidant status (TOS), total antioxidant activity (TAC), and oxidative stress index (OSI) for wild-caught and captive mole-rats, the highveld mole-rat, *Cryptomys hottentotus pretoriae*, and the common mole-rat, *C. h. hottentotus* in response to subspecies, status (wild-caught/captive), the interaction between subspecies and status, and the estimated resting metabolic rate (ERMR).

	TOS		TAC		OSI	
	t	*p*	t	*p*	t	*p*
Species	1.139	0.26	nr	nr	−0.459	0.65
Status	0.175	0.86	6.169	<0.001 *	3.344	0.001 *
Species × Status	3.631	<0.001 *	nr	nr	4.244	<0.001 *
ERMR	nr	nr	nr	nr	nr	nr

nr: Variable not retained in the final model. An * signifies significance.

**Table 4:** The best generalised linear model output for plasma oxidative markers, namely, the total oxidant status (TOS), total antioxidant activity (TAC), and oxidative stress index (OSI) for wild-caught mole-rats, namely, the highveld mole-rat, *Cryptomys hottentotus pretoriae*, the common mole-rat, *C. h. hottentotus* and the Natal mole-rat, *C. h. natalensis*, in response to subspecies and the estimated resting metabolic rate (ERMR).

	TOS		TAC		OSI	
	z	*p*	z	*p*	t	*p*
Subspecies CHN	5.202	<0.0001	6.063	<0.0001	2.083	0.037
Subspecies CHP	3.906	<0.0001	0.885	0.38	3.773	<0.001
ERMR	nr	nr	nr	nr	nr	nr

nr: Variable not retained in the final model.

## Data Availability

Data are contained within the article or Supplementary Materials.

## Supplementary Materials

The following supporting information can be downloaded at: https://www.mdpi.com/article/10.3390/antiox12081486/s1. File S1: The family of Bathyergidae, their distribution, their life history, reproductive structure, reproductive suppression and oxidative ecology; File S2: Data.
