# Supplementary material for: The Evolution and Ecology of Oxidative and Antioxidant Status: A Comparative Approach in African Mole-Rats
Source: Antioxidants (Basel). 2023 Jul 25;12(8):1486. doi: 10.3390/antiox12081486 (PMC10451868; doi:10.3390/antiox12081486)
Supplement: Supplementary file 1 [file antioxidants-12-01486-s001.zip › Supplementary S1 17072023 FINAL.pdf]

Supplementary

The Bathyergidae family

The African mole-rats of the family Bathyergidae (Rodentia) have some 22 described species from six genera, with a wide distribution (reviewed by Šumbera [1]) [2,3], and are endemic to sub-Sahara Africa. The social groups include three genera, *Heterocephalus*, a monotypic genus in the Horn of Africa [2,3], *Fukomys,* in savannah habitats in tropical Africa, and *Cryptomys,* limited to the subtropics and Mediterranean Southern African region [2,4]. The solitary species include three genera, namely *Bathyergus,* which occurs in the western and/or northern Cape of South Africa and *Georychus,* which occurs in forested and savannah regions across the coastal regions of Western Cape and Eastern Cape provinces in South Africa [4,5]. The last social species include another monotypic genus, *Heliophobius,* which occurs mainly in Eastern Africa [1].

Life history

Importantly, bathyergids are subterranean and/or fossorial, meaning they live and forage almost exclusively below ground [1,6-8]. Despite the benefits of microclimate stability and a lack of predation pressure that a subterranean environment provides, it also presents physiological challenges such as hypoxia, hypercapnia, high relative humidity, and little or no exposure to sunlight [1,6-12]. These animals pay a high energy cost to find food due to digging through soil [13,14]. Digging can also increase the risk of overheating, as high humidities in the burrow complicate heat dissipation [6,15,16], contributing to exercise-induced hyperthermia and possible oxidative damage [17]. The ecological conditions of an underground lifestyle, and adaptations to this niche, such as differences in respiration [7,8,18], may have resulted in the concurrent evolution of factors that may also mitigate oxidative damage [19,20].

Mole-rat social reproductive structure

Bathyergids also show a broad spectrum of sociality from solitary to social, the species exhibiting social or eusocial behaviour, a few dominant animals monopolise reproduction, with one breeding female and one to three larger males responsible for reproduction [21]. The remaining colony members (referred to as non-breeders). Social species typically show a reproductive division of labour, cooperative care of the young, and overlapping generations [22]. Sherman *et al.* [23] criteria include bimodality, resulting in a high reproductive skew among breeding females and/or males where the maximum lifetime reproductive success of breeders versus non-breeders (helpers) is far greater than that of female breeders versus helpers in cooperatively breeding vertebrate societies. In this definition by Sherman *et al.* [23], the proportion of non-breeding animals obtaining reproductive status during their lifetime can be used as a stricter measure of eusociality. As such, currently, only the Damaraland mole-rat, *F. damarensis* and the naked mole-rat *H. glaber* are considered eusocial due to a lack of empirical data pertaining to lifetime reproductive success for the Ansells mole-rat *F. anselli* and Giant mole-rat or Mechows mole-rat, *F. mechowii*as, they do not fit the stricter conditions for the claim of eusociality according to the definition of Sherman *et al.* [23].

Mole-rat reproductive suppression and lifting of suppression

In order to control the breeding of non-breeders, breeders of social mole-rat colonies utilise natural reproductive suppression mechanisms either through behavioural [24-26], physiological [27-32] and/or both processes [24]. Typically, behavioural suppression includes incest avoidance and dominance, with blocked breeding attempts, where hormones do not influence reproduction [24,26]. Physiological suppression, however, has come in several forms and is dependent on species, either through prolactin [27] or through mediating the gonadotropin-releasing hormone (GnRH) response [32,33]. Non-breeding colony members can disperse from their natal colonies during periods of high rainfall when the soil characteristics are optimal for excavation and digging [34-36], where dispersal can result in the relaxation or lifting of reproductive suppression [35,37-39]. Their reproductive system may likely be relevant to the differences observed in oxidative markers and ageing [4,40,41]. For

example, in the genus *Fukomys* and *Heterocephalus,* breeding animals live longer than their non-breeding counterparts [42-45].

References

1. Šumbera, R. Thermal biology of a strictly subterranean mammalian family, the African mole-rats (Bathyergidae, Rodentia)—A review. *J. Therm. Biol.* **2019**, *79*, 166–189.
2. Faulkes, C.G.; Bennett, N.C. African mole-rats: Social and ecological diversity. *Rodent Soc. Ecol. Evol. Perspect.* **2007**, 427–437.
3. Holtze, S.; Braude, S.; Lemma, A.; Koch, R.; Morhart, M.; Szafranski, K.; Platzer, M.; Alemayehu, F.; Goeritz, F.; Hildebrandt, T.B. The microenvironment of naked mole-rat burrows in East Africa. *Afr. J. Ecol.* **2018**, *56*, 279–289.
4. Visser, J.H.; Bennett, N.C.; van Vuuren, B.J. Phylogeny and biogeography of the African Bathyergidae: A review of patterns and processes. *PeerJ* **2019**, *7*, e7730.
5. Bennett, N.C.; Faulkes, C.G.; Hart, L.; Jarvis, J.U. *Bathyergus suillus* (Rodentia: Bathyergidae). *Mamm. Species* **2009**, *828*, 1-7.
6. Burda, H.; Šumbera, R.; Begall, S. Microclimate in burrows of subterranean rodents—Revisited. In *Subterranean Rodents*; Springer: Berlin/Hiedelberg, Germany, 2007; pp. 21–33.
7. Begall, S.; Burda, H.; Schleich, C.E. *Subterranean Rodents: News from Underground*; Springer: Berlin/Hiedelberg, Germany, 2007.
8. Lacey, E.A. *Life Underground: The Biology of Subterranean Rodents*; University of Chicago Press: Chicago, IL, USA, 2000.
9. Nevo, E. Adaptive convergence and divergence of subterranean mammals. *Annu. Rev. Ecol. Syst.* **1979**, *10*, 269–308.
10. Roper, T.; Bennett, N.; Conradt, L.; Molteno, A. Environmental conditions in burrows of two species of African mole-rat, *Georhychus capensis* and *Cryptomys damarensis*. *J. Zool.* **2001**, *254*, 101–107.
11. Ivy, C.M.; Sprenger, R.J.; Bennett, N.C.; van Jaarsveld, B.; Hart, D.W.; Kirby, A.M.; Yaghoubi, D.; Storey, K.B.; Milsom, W.K.; Pamenter, M.E. The hypoxia tolerance of eight related African mole-rat species rivals that of naked mole-rats, despite divergent ventilatory and metabolic strategies in severe hypoxia. *Acta Physiol.* **2020**, *228*, e13436.
12. Logan, S.M.; Szereszewski, K.E.; Bennett, N.C.; Hart, D.W.; Van Jaarsveld, B.; Pamenter, M.E.; Storey, K.B. The brains of six African mole-rat species show divergent responses to hypoxia. *J. Exp. Biol.* **2020**, *223*, jeb215905.
13. Lovegrove, B. The cost of burrowing by the social mole rats (Bathyergidae) Cryptomys damarensis and Heterocephalus glaber: the role of soil moisture. *Physiol. Zool.* **1989**, *62*, 449–469.
14. Vleck, D. The energy cost of burrowing by the pocket gopher Thomomys bottae. *Physiol. Zool.* **1979**, *52*, 122–136.
15. Wallace, K.M.; van Jaarsveld, B.; Bennett, N.C.; Hart, D.W. The joint effect of micro-and macro-climate on the thermoregulation and heat dissipation of two African mole-rat (Bathyergidae) sub-species, *Cryptomys hottentotus* mahali and C. h. pretoriae. *J. Therm. Biol.* **2021**, *99*, 103025.
16. Hart, D.W.; van Jaarsveld, B.; Lasch, K.G.; Grenfell, K.L.; Oosthuizen, M.K.; Bennett, N.C. Ambient Temperature as a Strong Zeitgeber of Circadian Rhythms in Response to Temperature Sensitivity and Poor Heat Dissipation Abilities in Subterranean African Mole-Rats. *J. Biol. Rhythm.* **2021**, *36*, 461–469.
17. Jacobs, P.; Finn, K.T.; van Vuuren, A.K.J.; Suess, T.; Hart, D.W.; Bennett, N.C. Defining the link between oxidative stress, behavioural reproductive suppression and heterothermy in the Natal mole-rat (Cryptomys hottentotus natalensis). *Comp. Biochem. Physiol. Part B: Biochem. Mol. Biol.* **2022**, *261*, 110753.
18. Nevo, E.; Reig, O. Evolution of subterranean mammals at the organismal and molecular levels. In *Proceedings of the 5h International Theriological Congress, Rome, Italy, 22–29 August 1989*; Wiley-Liss: Hoboken, NJ, USA, 1990.
19. Fang, X.; Seim, I.; Huang, Z.; Gerashchenko, M.V.; Xiong, Z.; Turanov, A.A.; Zhu, Y.; Lobanov, A.V.; Fan, D.; Yim, S.H. Adaptations to a subterranean environment and longevity revealed by the analysis of mole rat genomes. *Cell Rep.* **2014**, *8*, 1354–1364.
20. Selman, C.; Blount, J.D.; Nussey, D.H.; Speakman, J.R. Oxidative damage, ageing, and life-history evolution: where now? *Trends Ecol. Evol.* **2012**, *27*, 570–577.
21. Bennett, N.C.; Faulkes, C.G. *African Mole-Rats: Ecology and Eusociality*; Cambridge University Press: Cambridge, MA, USA, 2000.
22. Michener, C.D. Comparative social behavior of bees. *Annu. Rev. Entomol.* **1969**, *14*, 299–342.
23. Sherman, P.W.; Lacey, E.A.; Reeve, H.K.; Keller, L. The eusociality continuum. *Behav. Ecol.* **1995**, *6*, 102–108.

24. Bennett, N.C.; Faulkes, C.G.; Molteno, A.J. Reproductive suppression in subordinate, non-breeding female Damaraland mole-rats: two components to a lifetime of socially induced infertility. *Proc. Biol. Sci.* **1996**, *263*, 1599–1603. https://doi.org/10.1098/rspb.1996.0234.
25. Lutermann, H.; Young, A.J.; Bennett, N.C. Reproductive status and testosterone among females in cooperative mole-rat societies. *Gen. Comp. Endocrinol.* **2013**, *187*, 60–65.
26. Oosthuizen, M.; Bennett, N.; Lutermann, H.; Coen, C. Reproductive suppression and the seasonality of reproduction in the social Natal mole-rat (*Cryptomys hottentotus* natalensis). *Gen. Comp. Endocrinol.* **2008**, *159*, 236–240.
27. Bennett, N.C.; Ganswindt, A.; Ganswindt, S.B.; Jarvis, J.; Zöttl, M.; Faulkes, C. Evidence for contrasting roles for prolactin in eusocial naked mole-rats, Heterocephalus glaber and Damaraland mole-rats, *Fukomys damarensis*. *Biol. Lett.* **2018**, *14*, 20180150.
28. Medger, K.; Bennett, N.C.; Ganswindt, S.B.; Ganswindt, A.; Hart, D.W. Changes in prolactin, cortisol and testosterone concentrations during queen succession in a colony of naked mole-rats (*Heterocephalus glaber*): A case study. *Sci. Nat.* **2019**, *106*, 26.
29. Blecher, A.S.; Bennett, N.C.; Medger, K.; Hagenah, N.; Ganswindt, A.; Oosthuizen, M.K. Effect of colony disruption and social isolation on naked mole-rat endocrine correlates. *Gen. Comp. Endocrinol.* **2020**, *295*, 113520.
30. Hart, D.; van Vuuren, A.J.; Erasmus, A.; Süess, T.; Hagenah, N.; Ganswindt, A.; Bennett, N. The endocrine control of reproductive suppression in an aseasonally breeding social subterranean rodent, the Mahali mole-rat (*Cryptomys hottentotus* mahali). *Horm. Behav.* **2022**, *142*, 105155.
31. Young, A.J.; Oosthuizen, M.K.; Lutermann, H.; Bennett, N.C. Physiological suppression eases in Damaraland mole-rat societies when ecological constraints on dispersal are relaxed. *Horm. Behav.* **2010**, *57*, 177–183.
32. Van der Walt, L.; Bennett, N.; Schoeman, S. Reproductive suppression and pituitary sensitivity to exogenous GnRH in the highveld mole-rat (*Cryptomys hottentotus* pretoriae). *J. Zool.* **2001**, *254*, 177–184.
33. Bennett, N.; Jarvis, J.; Faulkes, C.; Millar, R. LH responses to single doses of exogenous GnRH by freshly captured Damaraland mole-rats, Cryptomys damarensis. *Reproduction* **1993**, *99*, 81–86.
34. Jarvis, J.U.; O'Riain, M.J.; Bennett, N.C.; Sherman, P.W. Mammalian eusociality: A family affair. *Trends Ecol. Evol.* **1994**, *9*, 47–51.
35. Molteno, A.; Bennett, N. Rainfall, dispersal and reproductive inhibition in eusocial Damaraland mole-rats (Cryptomys damarensis). *J. Zool.* **2002**, *256*, 445–448.
36. Scantlebury, M.; Speakman, J.R.; Oosthuizen, M.K.; Roper, T.J.; Bennett, N.C. Energetics reveals physiologically distinct castes in a eusocial mammal. *Nature* **2006**, *440*, 795–797.
37. Spinks, A.; Van der Horst, G.; Bennett, N. Influence of breeding season and reproductive status on male reproductive characteristics in the common mole-rat, *Cryptomys hottentotus* hottentotus. *Reproduction* **1997**, *109*, 79–86.
38. Spinks, A.; Bennett, N.; Jarvis, J. Regulation of reproduction in female common mole-rats (Cryptomys hottentotus hottentotus): the effects of breeding season and reproductive status. *J. Zool.* **1999**, *248*, 161–168.
39. Janse van Rensburg, L. The reproductive biology of Cryptomys hottentotus pretoriae (Rodentia: Bathyergidae); University of Pretoria: Pretoria, South Africa, 2006.
40. Jacobs, P.J.; Hart, D.W.; Suess, T.; Janse van Vuuren, A.K.; Bennett, N.C. The Cost of Reproduction in a Cooperatively Breeding Mammal: Consequences of Seasonal Variation in Rainfall, Reproduction, and Reproductive Suppression. *Front. Physiol.* **2021**, *12*, 2116.
41. Jacobs, P.J.; Hart, D.W.; Bennett, N.C. Plasma oxidative stress in reproduction of two eusocial African mole-rat species, the naked mole-rat and the Damaraland mole-rat. *Front. Zool.* **2021**, *18*, 45. https://doi.org/10.1186/s12983-021-00430-z.
42. Dammann, P. Slow aging in mammals—Lessons from African mole-rats and bats. In *Seminars in Cell & Developmental Biology*; Academic Press: Cambridge, MA, USA, 2017; pp. 154–163.
43. Dammann, P.; Šumbera, R.; Maßmann, C.; Scherag, A.; Burda, H. Extended longevity of reproductives appears to be common in Fukomys mole-rats (Rodentia, Bathyergidae). *PLoS ONE* **2011**, *6*, e18757.
44. Schmidt, C.M.; Blount, J.D.; Bennett, N.C. Reproduction is associated with a tissue-dependent reduction of oxidative stress in eusocial female Damaraland mole-rats (*Fukomys damarensis*). *PLoS ONE* **2014**, *9*, e103286.
45. Schmidt, C.M.; Jarvis, J.U.; Bennett, N.C. The long-lived queen: reproduction and longevity in female eusocial Damaraland mole-rats (*Fukomys damarensis*). *Afr. Zool.* **2013**, *48*, 193–196.

Disclaimer/Publisher's Note: The statements, opinions and data contained in all publications are solely those of the individual author(s) and contributor(s) and not of MDPI and/or the editor(s). MDPI and/or the editor(s) disclaim responsibility for any injury to people or property resulting from any ideas, methods, instructions or products referred to in the content.
